# Modifications of the respiratory chain of *Bacillus licheniformis* as an alkalophilic and cyanide-degrading microorganism

**DOI:** 10.1007/s10863-024-10041-y

**Published:** 2024-11-05

**Authors:** Daniel Uribe-Ramírez, Lucero Romero-Aguilar, Héctor Vázquez-Meza, Eliseo Cristiani-Urbina, Juan Pablo Pardo

**Affiliations:** 1 Departamento de Ingeniería Bioquímica, Escuela Nacional de Ciencias Biológicas, Instituto Politécnico Nacional, Av. Wilfrido Massieu s/n, Unidad Profesional Adolfo López Mateos, Gustavo A. Madero, Ciudad de México, 07738 México; 2 Departamento de Bioquímica, Facultad de Medicina, Universidad Nacional Autónoma de México, Circuito Interior S/N, Ciudad Universitaria, Coyoacán, Ciudad de México, 04510 México

**Keywords:** *Bacillus licheniformis*, Respiratory chain, Cyanide, Quinol oxidase, NAD(P)H dehydrogenase

## Abstract

*Bacillus licheniformis* can use cyanide as a nitrogen source for its growth. However, it can also carry out aerobic respiration in the presence of this compound, a classic inhibitor of mammalian cytochrome c oxidase, indicating that *B. licheniformis* has a branched respiratory chain with various terminal oxidases. Here, we studied the modifications in the respiratory chain of *B. licheniformis* when cells were cultured in Nutrient Broth, an alkaline medium with ammonium, or an alkaline medium with cyanide. Then, we measured oxygen consumption in intact cells and membranes, enzyme activities, carried out 1D and 2D-BN-PAGE, followed by mass spectrometry analysis of BN-PAGE bands associated with NADH, NADPH, and succinate dehydrogenase activities. We found that cell growth was favored in a nutrient medium than in an alkaline medium with cyanide. In parallel, respiratory activity progressively decreased in cells cultured in the rich medium, alkaline medium with ammonium, and the lowest activity was in the cells growing in the alkaline medium with cyanide. *B. licheniformis* membranes contain NADH, NADPH, and succinate dehydrogenases, and the proteomic analysis detected the nitrate reductase and the bc, caa3, aa3, and bd complexes. The succinate dehydrogenase migrated with a molecular mass of 375 kDa, indicating its association with the nitrate reductase (115 kDa + 241 kDa, respectively). The NADH dehydrogenase of *B. licheniformis* forms aggregates of different molecular mass.

## Introduction

*Bacillus licheniformis*, a Gram-positive bacterium, is recognized as a safe (GRAS) microorganism with broad potential in many industrial applications such as food, pharmaceuticals, and environmental management (Muras et al. 2021; He et al. 2023). In this context, industrial wastewater contains a diversity of contaminants, like phenols, azo dyes, hydrocarbons, pesticides, heavy metals, and cyanide, among others (Bharagava et al. 2020; Garg et al. 2022). *B. licheniformis* has been used to improve the nutrient content of the soil and reduce contaminants, being a reliable management option (He et al. 2023). Moreover, *B. licheniformis* degrades crude oil, benzo(a)pyrene, paracetamol, and cyanide (Khanpour-Alikelayeh et al. 2020; Muras et al. 2021; Chopra and Kumar 2023; Pan et al. 2023; Uribe-Ramírez et al. 2024).

Using cyanide in processes such as gold mining generates high levels of liquid waste containing cyanide (Rangel-González et al. 2024). In addition, cyanide is required for many industrial applications, including the production of nylon, plastics, adhesives, cosmetics, medicines, fire retardants, anti-caking additives, and road salts (Luque-Almagro et al. 2016). Cyanide treatment systems are needed to eliminate potential toxicity issues related to the health of humans, wildlife, waterfowl, or aquatic life (Botz et al. 2016; Malmir et al. 2022). Different treatments have been used to remove cyanide from wastewater. However, biotechnological treatment is the most promising, using microorganisms that can break down the cyanide molecule and use it as a source of carbon or nitrogen for their metabolism (Ojaghi et al. 2018; Razanamahandry et al. 2019). Some of the microorganisms that have been used are *Pseudomonas putida* with a cyanide removal efficiency of 83% at an initial concentration of 3 mM KCN (Moradkhani et al. 2018), a mixed culture of *Bacillus* that degraded with 99% efficiency at an initial concentration of 19.2 mM cyanide (Mekuto et al. 2015), *Bacillus subtillis* degraded 87% of an initial KCN concentration of 7.68 mM (Rosario et al. 2023), and finally, *Bacillus licheniformis* which was reported with resistance to cyanide of up to 57.7 mM, having consumption of 32 mM (Uribe-Ramírez et al. 2024). It is important to thoroughly explore the mechanisms that give microorganisms resistance to this toxic compound.

Cyanide is present in many compounds, although not all of them are lethal. Hydrogen cyanide (HCN), sodium cyanide (NaCN), and potassium cyanide (KCN) are highly toxic, but nitriles containing the cyanide group are less toxic (Muderawan et al. 2023). The main effect of cyanide is the inhibition of mitochondrial cytochrome c oxidase, resulting in the inhibition of aerobic respiration (Gracia and Shepherd 2004).

The mitochondrial respiratory chain in mammals consists of four protein complexes: complex I (NADH: ubiquinone oxidoreductase), complex II (succinate: ubiquinone oxidoreductase), complex III (ubiquinol: ferricytochrome c oxidoreductase), and complex IV (ferrocytochrome c: oxygen oxidoreductase or cytochrome c oxidase). In addition, there are two mobile electron carriers: ubiquinone and cytochrome c, located in the inner mitochondrial membrane and the intermembrane space, respectively (Cooper and Clark 1994). The mitochondrial respiratory chain transfers electrons from different substrates to oxygen, the final electron acceptor, coupled to the generation of a proton electrochemical gradient (Guan et al. 2022).

Unlike the electron transport chain of mammalian mitochondria, bacteria have branched respiratory chains, which can use different electron transfer routes toward the oxygen molecule depending on the growth conditions (Kaila and Wikström 2021). The function of the respiratory chain in aerobic prokaryotic microorganisms and mitochondria is the same: to build up a gradient of protons, which is then used to synthesize ATP (Magalon et al. 2012). This flexibility of the energy-generating machinery is essential in adapting free-living bacteria, such as *B. subtilis*, to variations in oxygen and nutrient supply, a common feature in their natural environment (Winstedt et al. 1998). The entry point of electrons into the *B. licheniformis* respiratory chain is given by different dehydrogenases (succinate, NADH) that transfer electrons from the specific substrates to quinone. In turn, electrons are transferred to oxygen through different oxidases (Melo and Teixeira 2016).

**Fig. 1:**
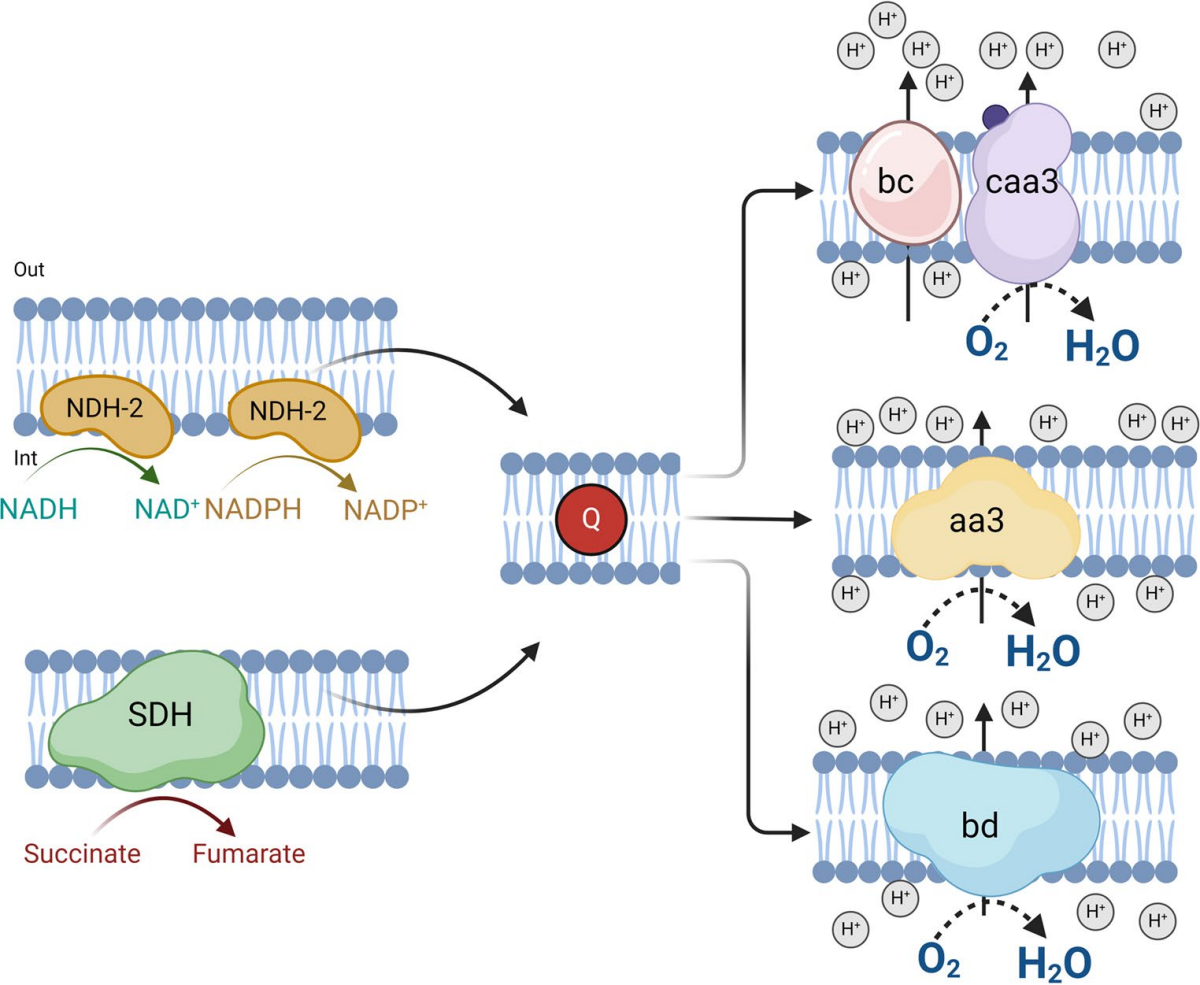
Respiratory chain of *Bacillus licheniformis*. NADH dehydrogenase (NDH-2), succinate dehydrogenase (SDH), quinone (Q), cytochrome bc (bc), caa3 (ferrocytochrome c: oxygen oxidoreductase), aa3 (quinol: oxygen oxidoreductase) and bd (quinol: oxygen oxidoreductase)

Searching the genome of *Bacillus licheniformis* ATCC14580, we found the genes encoding for proteins of the respiratory chain (Fig. 1). To carry out aerobic respiration, *B. licheniformis* uses a branched electron transport chain composed of alternative NADH dehydrogenases (NDH-2). These monotopic flavoproteins catalyze the oxidation of NADH and the reduction of quinone. Their location on the cytoplasmic surface of the bacterial membrane allows them to have access to the membrane-bound quinone. Since these enzymes do not have transmembrane segments, they do not translocate protons across the membrane (Björklöf et al. 2000; Lencina et al. 2018). *B. licheniformis* also has a succinate dehydrogenase containing two hemes (SDH, succinate: quinone oxidoreductase (electrogenic, proton-motive force generating) that catalyzes the two-electron reduction of quinone by succinate (Azarkina and Konstantinov 2002). It is worth mentioning that *B. licheniformis* lacks complex I.

The cytochrome bc1 of *Bacillus* species and Actinobacteria has features common to cytochrome b6f of chloroplasts and cyanobacteria, rather than the canonical bc1 complex (complex III) of mitochondria and many aerobic Gram-negative bacteria (Hederstedt 2021). For this reason, this cytochrome will be named bc, as described by Sousa et al. 2013. It is an integral membrane enzyme that catalyzes the transfer of electrons from quinol to a type c cytochrome, and like the mitochondrial complex III, it is inhibited by antimycin A (Sousa et al. 2013). Coupled with the redox reaction, protons are translocated across the membrane, generating a proton electrochemical gradient (Yu et al. 1995). The cytochrome bc complex donates electrons from quinol to cytochrome caa3. The two small cytochromes c, c-550 and c-551, facilitate the transfer of electrons between the cytochrome c domain of the bc complex and that of cytochrome caa3 (Hederstedt 2021). These two cytochromes in *B. subtilis* are anchored to the cytoplasmic side of the membrane: cytochrome c-550 by an N-terminal transmembrane segment and cytochrome c-551 by a diacylglycerol tail (Sousa et al. 2013). The small cytochromes c are not free in the membrane but confined to the bc-caa3 super complex (Picón Garrido et al. 2022).

The search of the *B. licheniformis* genome indicates that this microorganism contains three types of terminal oxidases: a caa3 (ferrocytochrome-c: oxygen oxidoreductase), aa3 (quinol: oxygen oxidoreductase), and bd (quinol: oxygen oxidoreductase). The first one probably functions as the mitochondrial cytochrome c oxidase, while the last two use quinol (probably menaquinol) as substrate. Both a-type oxidases are members of the well-characterized superfamily of heme-copper terminal oxidases (Winstedt et al. 1998; Sousa et al. 2013), with similar structures and the capacity to pump protons out of cells driven by the redox reactions (Esposti 2020; Hederstedt 2021).

Cytochrome bd oxidases of bacterial and archaeal respiratory chains couple the redox reaction consisting of quinol oxidation and reduction of dioxygen with the transfer of protons across the membrane. Cytochrome bd oxidase shows a high affinity for dioxygen, allowing bacteria to grow under microaerobic conditions (Safarian et al. 2016; Theßeling et al. 2019). These enzymes protect against the toxicity of classical respiratory inhibitors, for example, nitric oxide (NO), cyanide (CN^−^), and hydrogen sulfide (H_2_S) (Borisov et al. 2021). The lower sensitivity of cytochrome bd oxidases to cyanide may be due to the lack of CuB and/or a high electron density in the central iron atom that originates from breaking the conjugated structure of the π electron in the haem dihydroxy chlorine ring (Forte et al. 2017).

In this study, we utilized bioinformatic approaches, measurements of cellular and plasma membrane oxygen consumption, one- and two-dimensional blue native electrophoresis, enzyme assays, and proteomic analysis to describe the performance of respiratory chain components in *B. licheniformis* when the bacterial cells were cultured in an alkaline mineral medium containing cyanide.

## Materials and methods

### Reagents

Reagents were obtained from JT Baker (Avantor Performance Materials, Inc., Xalostoc, Estado de México, Mexico) and Sigma-Aldrich (Sigma-Aldrich, Co., St. Louis, MO, USA).

### Cell culture

Two 2-L glass flasks containing 500 mL of Nutrient Broth (Nut) were inoculated with 5 mL of an overnight culture of *B. licheniformis* in Nutrient Broth, then the cells were grown for 24 h at 140 rpm, 25^o^ C, and then collected by centrifugation at 17,000 g for 10 min. The pellets were resuspended in minimal media pH 11 at an approximate concentration of 1 g L^− 1^ of biomass. The composition of the medium was the following: 100 mM phosphate buffer, MgSO_4_·7H_2_O, 0.018 mg L^− 1^; CoCl_2_, 0.013 mg L^− 1^; CaCl_2_, 0.004 mg L^− 1^; ZnSO_4_, 0.004 mg L^− 1^; Na_2_Mo_4_·2H_2_O, 0.002 mg L^− 1^; MnSO_4_·H_2_O, 0.01 mg L^− 1^; NiSO_4_·6H_2_O, 0.01 mg L^− 1^; CuSO_4_·7H_2_O, 0.01 mg L^− 1^; and FeCl_3_·6H_2_O, 0.005 mg L^− 1^, 6 g L^− 1^ sodium acetate was used as a carbon source and different nitrogen sources, 5.5 mM (NH_4_)_2_SO_4_ or 3.84 mM KCN. A sample was taken every 4 h, and the biomass (dry weight) was quantified. At the same time, growth in Nutrient Broth was conducted as a control.

### Obtaining membranes

Cells cultured in the three growth conditions were collected by centrifugation at 17,000 g for 10 min and washed in a 1:1 (v/v) ratio with PBS buffer (2.7 mM KCl, 137 mM NaCl, 10 mM KH_2_PO_4_, 10 mM K_2_HPO_4_, pH 7.4). The cell pellet was resuspended in PBS buffer in a ratio of 10 mL of buffer per 1 g of wet weight, and the suspension was supplemented with 2.7 mM MgCl_2_, 5 mM phenylmethylsulfonyl fluoride (PMSF), 40 mg mL^− 1^ DNAse and 10 mg mL^− 1^ lysozyme. The cells were disrupted with a Soniprep 150 cell disruptor in 10 sonication cycles of 1 min with 1 min rest in an ice-water bath. The suspension was centrifuged at 17,000 g for 10 min, and the supernatant was centrifuged at 100,000 g for 1 h at 4 °C. The membranes were resuspended in a small volume of PBS buffer.

### Oxygen consumption

Oxygen consumption by cells and their respective membranes was evaluated in a 1.5 mL chamber at 30 °C, using a Clark-type electrode connected to a YSI5300A biological oxygen monitor. The assays were carried out with cells (0.05 to 0.1 units of Abs) or membranes (0.5 mg protein/mL) in nutrient broth medium or respiration buffer (20 mM HEPES-KOH, 135 mM KCl, 5 mM MgCl_2_, 1 mM EGTA, 5 mM K_2_HPO_4_, pH 7). The inhibitors and their concentrations used in the experiments with cells were 1 mM KCN, 28.8 µM antimycin A, and 960 µM flavone. In the case of the membranes, 10 mM NADH was added as substrate, and the inhibitors used were 1 mM KCN, 4.8 µM antimycin A, and 184 µM flavone. KCN is an inhibitor of complex caa3, antimycin A of complex bc, and flavone of the alternative NADH dehydrogenases (NDH-2). Antimycin A and flavone were prepared in ethanol, so a control experiment was carried out in which oxygen consumption was recorded in the presence of only ethanol.

### Enzymatic activity

NDH-2 activity was determined according to Esparza-Perusquía et al. (2015) in a buffer containing 10 mM HEPES pH 7, 2 mM MgSO_4_, 100 µM NADH or NADPH, 100 µM 2,6 dichlorophenolindophenol (DCPIP), and 10–50 µL of the membrane preparation, in a final volume of 1 mL. The reduction of DCPIP was followed spectrophotometrically at 600 nm. The molar extinction coefficient at 600 nm of the oxidized DCPIP is 21 mM^− 1^ cm^− 1^. Specific activity will be reported as nmol of reduced DCPIP min^− 1^ (mg of protein)^−1^.

Succinate dehydrogenase activity was determined according to Esparza-Perusquía et al. (2015) in 10 mM HEPES pH 7, 2 mM MgSO_4_, 10 mM succinate, 100 µM DCPIP, and 10–50 µL of the membrane preparation in a final volume of 1 mL. The reduction of DCPIP was followed spectrophotometrically at 600 nm. The molar extinction coefficient at 600 nm of the oxidized DCPIP is 21 mM^− 1^ cm^− 1^. Specific activity will be reported as nmol of reduced DCPIP min^− 1^ (mg of protein)^−1^.

### Activity in 1D and 2D blue native gels

The proteins in *B. licheniformis* membranes were solubilized by suspending them in 200 µL of 50 mM Bis-Tris buffer and 500 mM 6 aminocaproic acid pH 7.0 and 12 µL of digitonin (50% stock solution) were added drop by drop, under agitation in a vortex, to reach a detergent/protein ratio of 2 (g/g). The mixture was incubated at 4 °C for 30 min and centrifuged at 100,000 g for 35 min at 4 °C. The supernatants were recovered and mixed with 10 µL of buffer containing 10% glycerol, 0.2% Coomassie Brilliant Blue G-250, and 20 mM 6-aminocaproic acid.

For electrophoresis in one dimension (1D), approximately 150 µg of protein per lane were loaded on a blue native gel (BN-PAGE) with a linear gradient of 4 to 10% polyacrylamide. The anode buffer solution contained 50 mM Bis-Tris/HCl, pH 7.0; the cathode buffer solution contained 50 mM tricine, 15 mM Bis-Tris, pH 7.0, and Coomassie Brilliant blue dye 0.02%.

For electrophoresis in the second dimension (2D BN-PAGE), a lane from the 1D BN-PAGE was incubated in a 0.02 or 0.1% solution of N-dodecyl-β-D-maltoside (DDM) and placed onto the 2D gel. Electrophoresis was performed at 4 °C with a voltage of 35 V for 12 h. Electrophoresis was stopped when the sharp line of the dye approached the front of the gel.

### Activities in the gel

#### NADH dehydrogenase

The activity of NADH dehydrogenase was determined in a solution containing 5 mg methyl thiazolyl diphenyl-tetrazolium bromide (MTT) and 3.75 mg of NADH in 10 mL of 10 mM Tris-HCl pH 7.4. After 1 h at 25 ºC, the reaction was stopped with a fixation solution (50% methanol, 10% acetic acid).

#### NADPH dehydrogenase

The activity of NADH dehydrogenase was determined in a solution containing 5 mg methyl thiazolyl diphenyl-tetrazolium bromide (MTT) and 1.16 mg of NADPH in 10 mL of 10 mM Tris-HCl pH 7.4. After 1 h at 25 ºC, the reaction was stopped with a fixation solution (50% methanol, 10% acetic acid).

#### Succinate dehydrogenase

The activity of succinate: MTT oxidoreductase was determined in a solution containing 100 mM sodium succinate, 1 mg phenazine methosulfate, 4.5 mM EDTA, and 20 mg MTT in 10 mL of 50 mM K_2_HPO_4_ (pH 7.4). The assays were carried out at 25º C for 1 h to develop the color, and then the reaction was stopped with the fixing solution.

#### Cytochrome caa3 oxidase activity

For complex of caa3, the gel was incubated at 25 ºC in 10 mL of 50 mM K_2_HPO_4_ (pH 7.2) with 10 mg of diaminobenzidine and 2 mg of horse heart cytochrome c. After 12 h of incubation in the reaction mixture, the gel was transferred to the fixation solution.

#### Coomassie stain

Coomassie blue staining was used to visualize the proteins in the gel. The gel was stained with a Coomassie solution (0.25% Coomassie Brillant Blue R-250 in 40% methanol and 7% acetic acid) for 3 h and destained with 5% methanol and 7% acetic acid.

#### Proteomics studies

The solubilized proteins from nutrient broth were subjected to 1D BN-PAGE, and one lane was used to detect bands with NADH dehydrogenase activity and a second one for succinate dehydrogenase. Four bands containing NADH-DH and one with succinate-DH activity were cut and sent to the Institut de recherches cliniques de Montreal, Montreal, Canada (https://www.ircm.qc.ca/en/mass-spectrometry-and-proteomics*)* for the proteomic study.

Proteins were quantified using the Lowry method (Lowry et al. 1951). Bovine serum albumin was used as standard.

## Results and discussion

### Growth of *B. licheniformis* in nutritive medium, minimal medium with ammonium, and minimal medium with cyanide

**Fig. 2:**
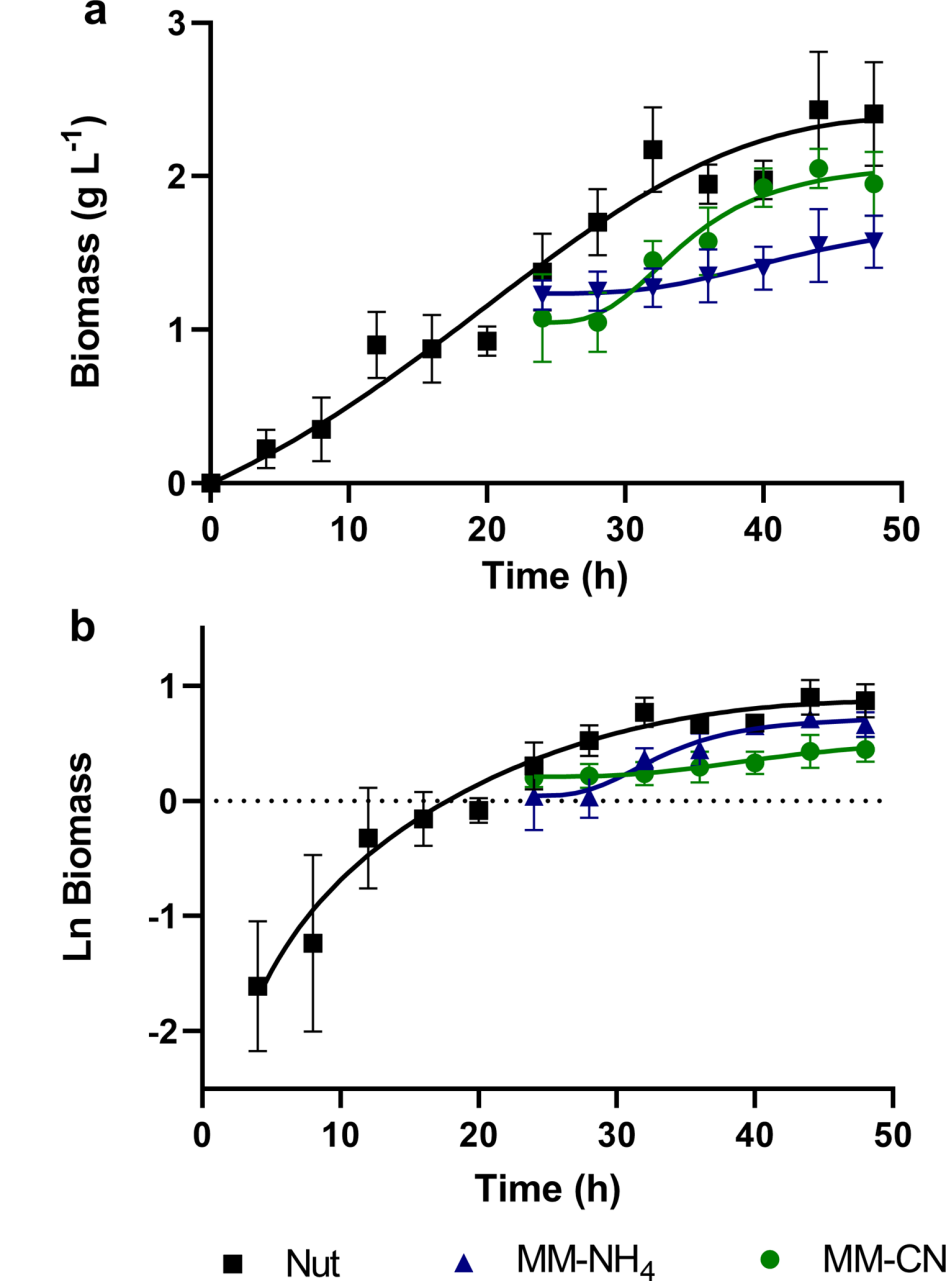
Growth of *Bacillus licheniformis* in different culture media at 25 ºC and 140 rpm. **a**) Growth under the 3 culture conditions, **b**) plot of the growth curves in semi log scale

It has been reported that several bacteria, including *B. licheniformis*, can use cyanide as a nitrogen source (Mekuto et al. 2013; Rosario et al. 2023; Cáceda Quiroz et al. 2023). The degradation of cyanide associated with the production of ammonia was observed in batch cultures of *B. licheniformis* (Uribe-Ramírez et al. 2024). To further study the effect of cyanide on the growth of *B. licheniformis*, the bacterium was cultured in three different media: Nutritive Broth (Nut), an alkaline mineral minimum medium that contained ammonium (MM-NH_4_), and an alkaline mineral minimum medium with cyanide (MM-CN). Figure 2 shows the growth profiles of *B. licheniformis* at 25 ºC for 48 h in the three conditions studied. The results indicated that *B. licheniformis* could grow in the 3 culture media with different growth rates and biomass yields.

Cells grown in Nut medium reached a biomass concentration of 2.3 g L^− 1^ after 48 h incubation. The growth curves for MM-NH_4_ and MM-CN media began with approximately 1 g L^− 1^ of biomass to get enough material for the membrane isolation. *B. licheniformis* grew in MM-NH_4_ medium with a maximum specific growth rate of 0.061 h^− 1^ and reached a final biomass concentration of 2 g L^− 1^, implying a net biomass production of 1 g L^− 1^. In the case of the MM-CN medium, there was a lag phase of approximately 12 h, followed by an exponential increase in biomass with a maximum specific growth rate of 0.016 h^− 1^, and a final biomass concentration of 1.6 g L^− 1^ (net biomass production of 0.6 g L^− 1^) was reached at the end of the experiment. The highest maximum specific growth rate (0.167 h^− 1^) was obtained with the Nut medium. These results agree with those reported for *Pseudomonas fluorescens* with the same two nitrogen sources assayed in this work (NH_4_^+^ and CN^−^), obtaining better growth when ammonium was used as the nitrogen source (Harris and Knowles 1983). An explanation for the lower growth in the MM-CN condition compared to MM-NH_4_ is that the conversion of CN^−^ into NH_4_^+^ is too slow, limiting the synthesis of biomolecules and the growth of the cells.

### Oxygen consumption by *B. licheniformis* cells

**Fig. 3:**
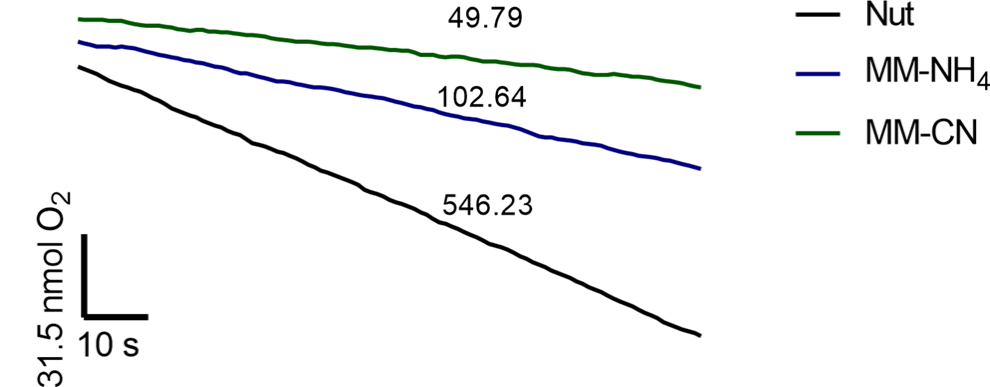
Oxygen consumption by *B. licheniformis* cells grown in the three different culture conditions assayed. The numbers in the figure indicate the oxygen consumption rates in nmol min^− 1^ (mg dry weight)^−1^

Oxygen consumption is an indicator of the metabolic status of the cell. A high respiratory rate indicates an abundance of respiratory enzymes and ATP synthase to support cell growth. Therefore, we studied the changes in oxygen consumption by *B. licheniformis* cultured in the three growth conditions tested (Fig. 3). The highest rate of oxygen consumption was observed in cells grown in Nut (546.23 nmol O_2_ min^− 1^ (mg dry weight)^−1^), followed by cells in MM-NH_4_ (102.64 nmol O_2_ min^− 1^ (mg dry weight)^−1^), and lastly by cells grown in MM-CN (49.74 nmol O_2_ min^− 1^ (mg dry weight)^−1^). The cell growth rate in the different media followed closely the respiratory rate. These results indicated a higher abundance of the respiratory elements in the Nut-cells.

To determine the composition of the respiratory chain of *B. licheniformis* grown in the three culture conditions, we used specific inhibitors that interact with the respiratory enzymes in the plasma membrane. Flavone is well known to inhibit alternative NDH-2 dehydrogenases, and antimycin A and KCN interact with complex bc and cytochrome c oxidase, respectively. It is worth mentioning that in our experiments, the respiratory chain was fed with NADH, showing that the entry point of the electrons will depend on the activity of alternative NADH dehydrogenases.

**Fig. 4:**
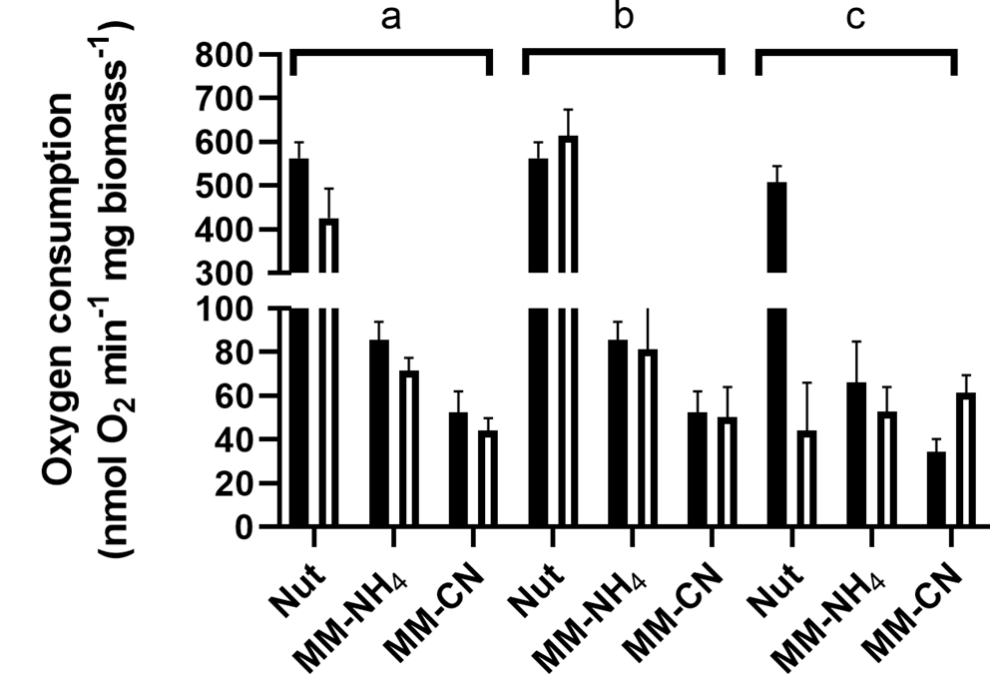
Effect of respiratory chain inhibitors on oxygen consumption of cells grown in Nut, MM-NH_4_, and MM-CN. Black bars represent oxygen consumption without an inhibitor, and empty bars represent oxygen consumption with an inhibitor. **a**) Flavone (960 µM), **b**) Antimycin A (28.8 µM), and **c**) KCN (1 mM)

Despite the high flavone concentration used in the assays, its inhibitory effect was small, at most 35% in the cells grown in the Nut medium. A smaller inhibition was observed for cells grown in MM-NH_4_ and MM-CN media (Fig. 4). Antimycin A did not affect the respiratory activity of the cells, regardless of the culture conditions (Fig. 4). Since antimycin A inhibits the bc complex, which is linked to the caa3 terminal oxidase, the results suggest the lack or minor amounts of these elements in the respiratory chain of *B. licheniformis*, at least under our culture conditions. For the cells grown in the Nut medium, cyanide inhibited respiration by around 90%. In contrast, for the cells cultivated in the MM-NH_4_ medium, the inhibitor did not significantly change their respiratory activity, and for the cells grown in the MM-CN medium, the addition of cyanide to the reaction medium increased oxygen consumption.

**Fig. 5:**
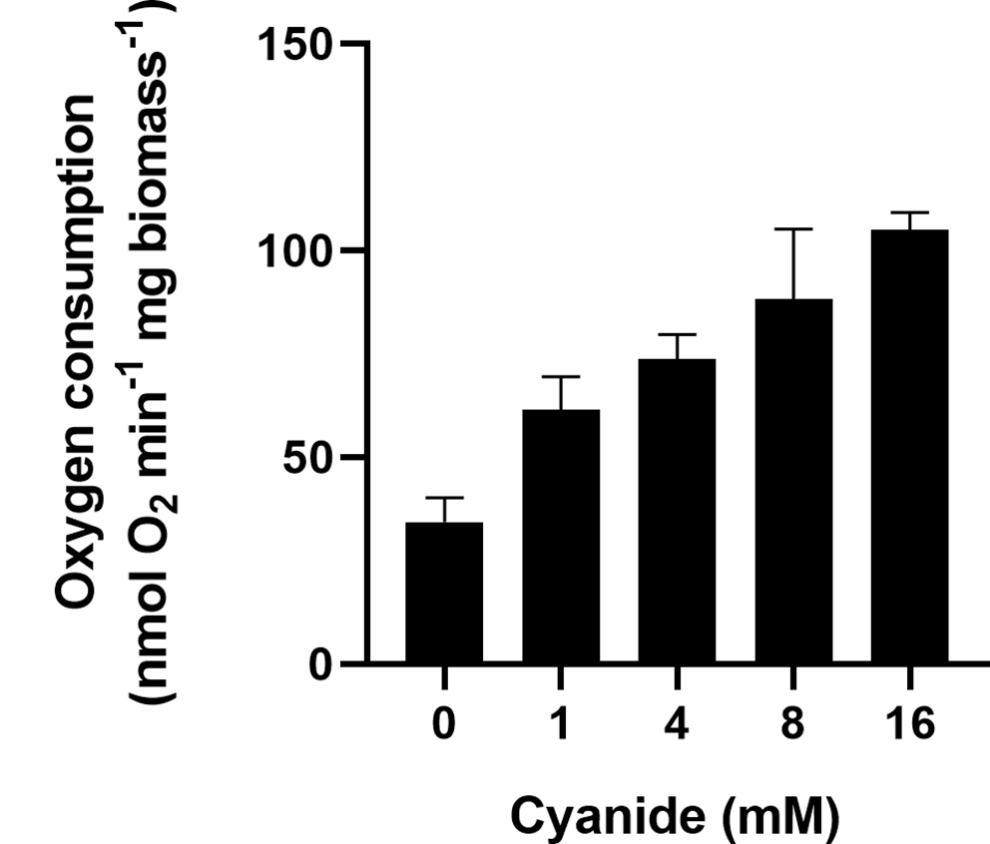
Oxygen consumption by cells grown in MM-CN at different potassium cyanide concentrations

To further characterize the activating effect of cyanide on the respiratory activity of whole MM-CN-grown cells, we studied oxygen consumption at different cyanide concentrations. Figure 5 shows that the respiratory activity of MM-CN-grown cells depends on the cyanide concentration. It was found that the Michaelis-Menten model describes the relationship between oxygen consumption and the cyanide concentration, with a Vm of 8.0 nmol min^− 1^ (mg dry biomass)^−1^ and a Km for cyanide of 3.1 mM. Given that some reactions in the cyanide assimilation pathway involve oxygen consumption (Alvillo-Rivera et al. 2021), the simplest explanation is that the increase in oxygen consumption is due to reactions involved in cyanide metabolism. The observation that cyanide stimulated oxygen consumption in the cytosolic fraction of MM-CN-grown cells (Table 1) supports this interpretation.

**Table 1:** Oxygen consumption by cell-free extracts from MM-CN-grown cells at different cyanide concentrations

Cyanide (mM)	Oxygen consumption (nmol O_2_ min^− 1^ (mg protein)^−1^)
4	36.8 ± 4.8
8	44.6 ± 2.6

It has been reported that an adequate supply of oxygen is important for the successful biodegradation of cyanide (Huertas et al. 2010). In this work, we demonstrate that the cyanide degradation metabolism of *Bacillus licheniformis* demands a high amount of oxygen.

### Oxygen consumption in *B. licheniformis* membranes

The activating effect of cyanide on cellular respiration is expected to be absent in isolated membrane preparations, as the enzymes responsible for cyanide metabolism are localized in the cytosol rather than the membrane fraction. Therefore, membranes were isolated from cells grown in the three conditions, and their oxygen consumption was measured using NADH as a substrate. In addition, we tested the three mentioned inhibitors to look for the different respiratory enzymes.

**Fig. 6:**
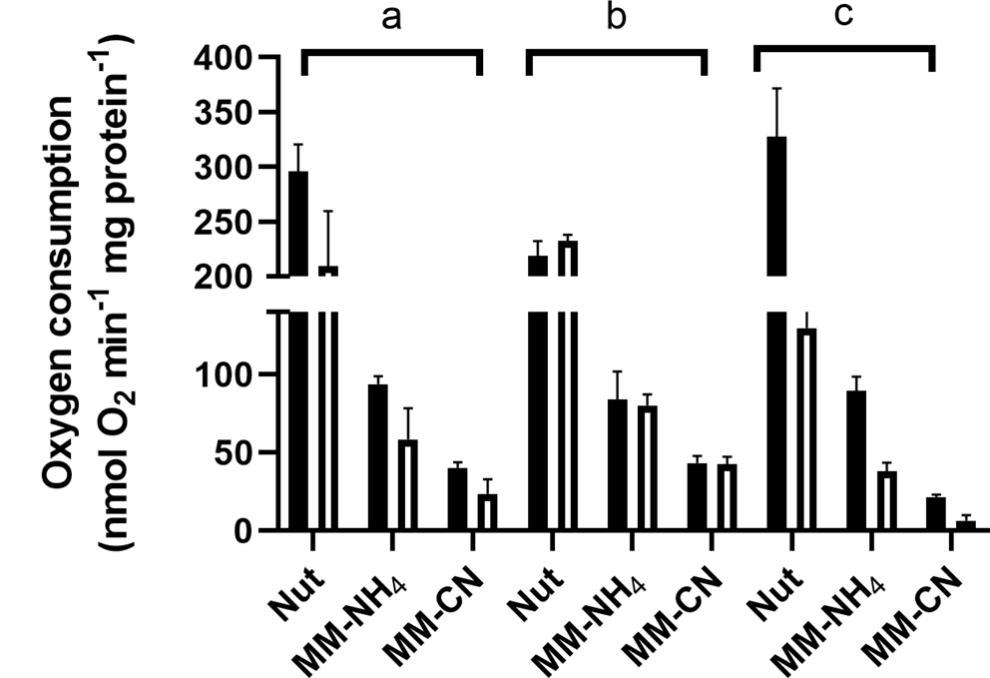
Oxygen consumption inhibition of cell membranes from Nut-, MM-NH_4_-, and MM-CN-grown cells. Black bars represent oxygen consumption without an inhibitor, and empty bars represent oxygen consumption with an inhibitor. **a**) Flavone (184 µM), **b**) Antimycin A (4.8 µM), and **c**) KCN (1 mM)

As shown in Fig. 6, oxygen consumption supported by NADH was highest in membranes from Nut-grown cells (290 nmol O_2_ min^− 1^ (mg protein)^−1^), followed by membranes from MM-NH_4_- (90 mol O_2_ min^− 1^ (mg protein)^−1^) and MM-CN (37 mol O_2_ min^− 1^ (mg protein)^−1^) grown cells. Similarly to the behavior of whole cells (Fig. 4), the inhibition by flavone was slight, regardless of the culture conditions. Oxygen consumption of membranes from Nut-grown cells was inhibited 30% by flavone and 40% in the other two conditions. Since the entrance of electrons to the respiratory chain depends on alternative NADH dehydrogenases, the partial inhibition of the oxygen uptake indicates that flavone might behave as a partial inhibitor, as described for the Ndi1 of *S. cerevisiae* (Velázquez and Pardo 2001). However, another explanation is that *B. licheniformis* NADH dehydrogenases are not inhibited by flavone. In agreement with the results obtained with whole cells, there was no inhibition by antimycin A, indicating that cytochrome bc and the caa3 terminal oxidase have a minor contribution to electron transport in this microorganism. Accordingly, the other two ubiquinol oxidases function as terminal oxidases.

Further information can be extracted from the conditions with cyanide. In agreement with the inhibition of oxygen consumption by whole cells, there was approximately a 60% inhibition by cyanide of membranes from Nut- and MM-NH_4_^_^grown cells. In contrast with the activating effect of cyanide in MM-CN-grown cells, in membranes from MM-CN-grown cells, there was 80% inhibition. Since the quinol oxidase aa3 is sensitive to high cyanide concentrations (Hill and Peterson 1998), the results suggest that 60 to 80% of the electron transport is due to the terminal oxidase aa3. In agreement with these data, it has been reported that cytochrome aa3 is mostly expressed in the respiratory chain of *B. subtilis* in the exponential phase (Winstedt et al. 1998). The remaining cyanide-insensitive (oxygen consumption in the presence of 1 mM cyanide) may originate from the bd-type terminal oxidases (Forte et al. 2017).

Several terminal oxidases have been described in *Pseudomonas aeruginosa*. It was reported that it has cytochrome bd, called Cio (cyanide-insensitive oxidase, encoded by the genes cioA and cioB), which is insensitive to cyanide (Luque-Almagro et al. 2011). Furthermore, *Bacillus* YN-2000 is a facultative alkaliphilic strain capable of growing in cyanide. The respiratory components of its plasma membrane were purified, and their oxygen consumption was measured and found to be 289 nmol O_2_ mg^− 1^ min^− 1^, which agrees with the oxygen consumption range of the *Bacillus licheniformis* cells used in this work. The oxygen consumption by membranes from *Bacillus* YN-2000 cells was 17.2 nmol O_2_ min^− 1^ (mg protein)^−1^ using NADH as a substrate and KCN (5 mM) as an inhibitor (Higashibata et al. 1998), comparable with what we report herein.

### Specific activities of the *B. licheniformis* dehydrogenases

Next, we assayed some of the enzymes that feed the respiratory chain with electrons. Since the genome of *B. licheniformis* predicts several alternative NADH dehydrogenases and there is the possibility that one of these enzymes may use NADPH as substrate, we measured the dehydrogenase activity in isolated membranes with NADH and NADPH.

**Fig. 7:**
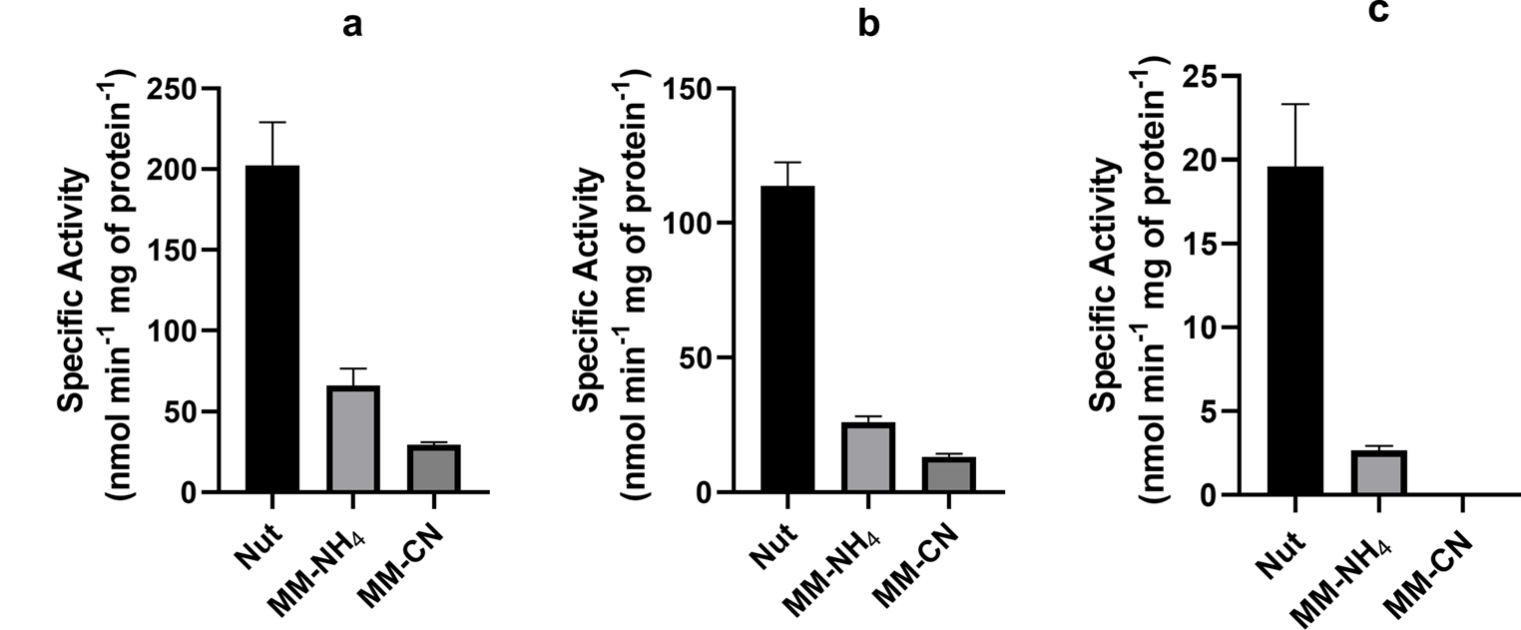
Specific activity of membrane dehydrogenases obtained from *Bacillus licheniformis* grown under the three culture conditions with different substrates. **a**) NADH, **b**) NADPH, and **c**) succinate

As shown in Fig. 7, membranes obtained from Nut-grown cells showed NADH dehydrogenase activity, which was around 200 nmol min^− 1^ (mg protein)^−1^, twice the activity found with NADPH (Fig. 7b). These two activities were much lower in the membranes from MM-NH_4_- and MM-CN-grown cells, indicating a smaller amount of these enzymes in cells cultured in these two media. The activity with succinate (20 nmol min^− 1^ (mg protein)^−1^) was tenfold lower than that of the NADH dehydrogenase in the Nut-grown cells (Fig. 7c). Furthermore, the succinate dehydrogenase activity was only detected in membranes obtained from cells cultured in Nut and MM-NH_4_ media.

Activities of 200 and 100 nmol min^− 1^ (mg protein)^−1^ were reported for NADH dehydrogenases in *Eikenella corrodens* and *Bacillus firmus*, respectively. The NADH dehydrogenase activities in both microorganisms were comparable to that in *B. licheniformis*. However, succinate dehydrogenase activities reported in *E. corrodens* and *B. firmus* (235 and 420 nmol min^− 1^ (mg protein)^−1^, respectively) are 10 to 20 times the activity found in *B. licheniformis* (Hicks and Krulwich 1995; Jaramillo-Lanchero et al. 2021). So far, the activity of NADH dehydrogenase is comparable in the three species, but the succinate dehydrogenase activity in *B. licheniformis* is too small compared to that in *E. corrodens* and *B. firmus*. The growth conditions can explain these differences. In our experiments, cells were cultured in a medium containing 0.6% sodium acetate as a carbon source, while a rich medium containing 1.5% casein enzymatic hydrolysate, 1.5% sodium formate, and 0.1% of the following amino acids: cysteine, proline, methionine, tryptophan, and serine were used to grow *E. corrodens* (Jaramillo-Lanchero et al. 2021). *B. firmus* was grown in 50 mM malate as the carbon source (Hicks and Krulwich 1995).

### Competition plot

**Fig. 8:**
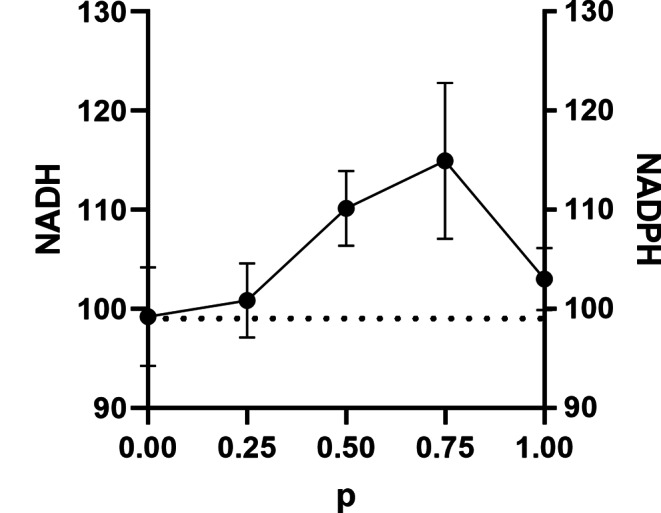
Competition plot for NADH and NADPH for NDH-2 of *Bacillus licheniformis* grown in Nut medium

Since there was a possibility that the same enzyme was acting on NADH and NADPH as substrates, we relied on a simple kinetic technique, the competition plot (Fig. 8) to differentiate between one site binding the two substrates as opposed to separate sites. The competition plot involves plotting the total reaction rate against the parameter p, which represents the relative concentrations of the two substrates. If the two substrates react at the same site, the competition plot shows a horizontal straight line, indicating that the total rate is independent of p (Chevillard et al. 1993). However, if the reactions occur at separate sites, the plot exhibits a curve with a maximum. When NADH and NADPH were varied, a curve with a maximum was obtained (Fig. 8), indicating that the *B. licheniformis* membrane contains at least two enzymes, one specific for NADH and the other for NADPH.

### Blue native gels of *B. licheniformis* respiratory enzymes

**Fig. 9:**
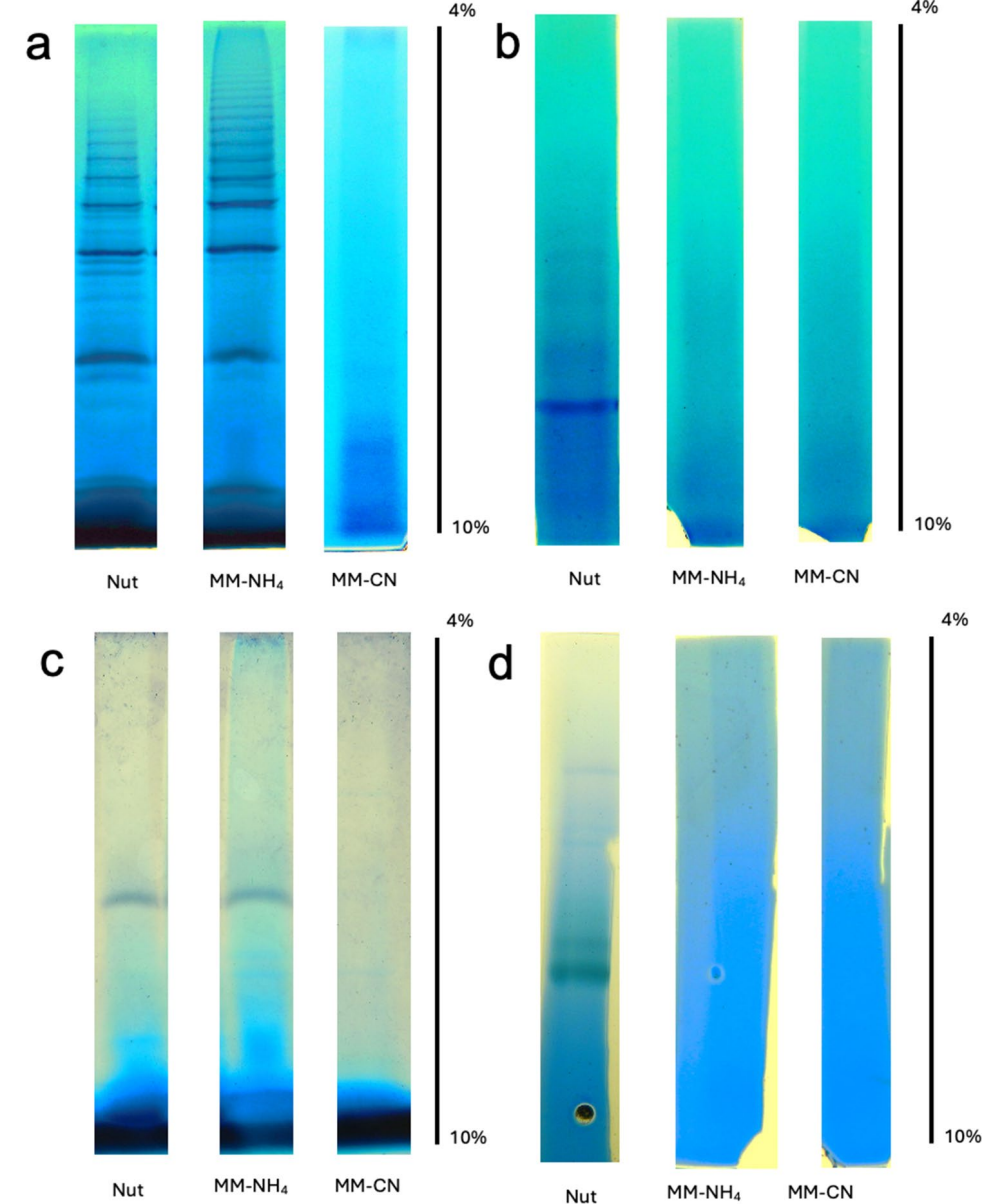
Gel activities of digitonin-solubilized respiratory complexes from *B. licheniformis* grown in Nut, MM-NH4, MM-CN media. BN-PAGE 1D in a linear gradient of 4 to 10% acrylamide. **a**) NADH dehydrogenase, **b**) NADPH dehydrogenase, **c**) succinate dehydrogenase, and **d**) cytochrome c oxidase

Up to now, the respiratory activity of *B. licheniformis* depends on the activity of NADH, NADPH, and succinate dehydrogenases, but how these enzymes behave inside the membrane is still an open question. Are they forming complexes with other proteins? Are their molecular masses in agreement with the values calculated from the genome? In this regard, the association of the *bc1* and *caa*_*3*_ complexes in *B. subtilis* and forming a bc1-caa3 supercomplex (García Montes De Oca et al. 2012), which might form respiratory strings in the membrane, has been reported (Picón Garrido et al. 2022). Additionally, a complex of the succinate dehydrogenase with the nitrate reductase has been proposed (Sousa et al. 2013). Therefore, the isolated membranes of *B. licheniformis* were incubated with digitonin to solubilize the proteins involved in the oxidative phosphorylation, then they were subjected to BN-PAGE, and the respiratory enzymes followed by specific in-gel activities. Approximately 150 µg of protein were loaded onto the gel for all conditions. Figure 9 shows the gel lanes with the activity of NADH dehydrogenase (Fig. 9a), NADPH dehydrogenase (Fig. 9b), succinate dehydrogenase (Fig. 9c), and cytochrome c oxidoreductase (Fig. 9d) for the three experimental conditions.

**Fig. 10:**
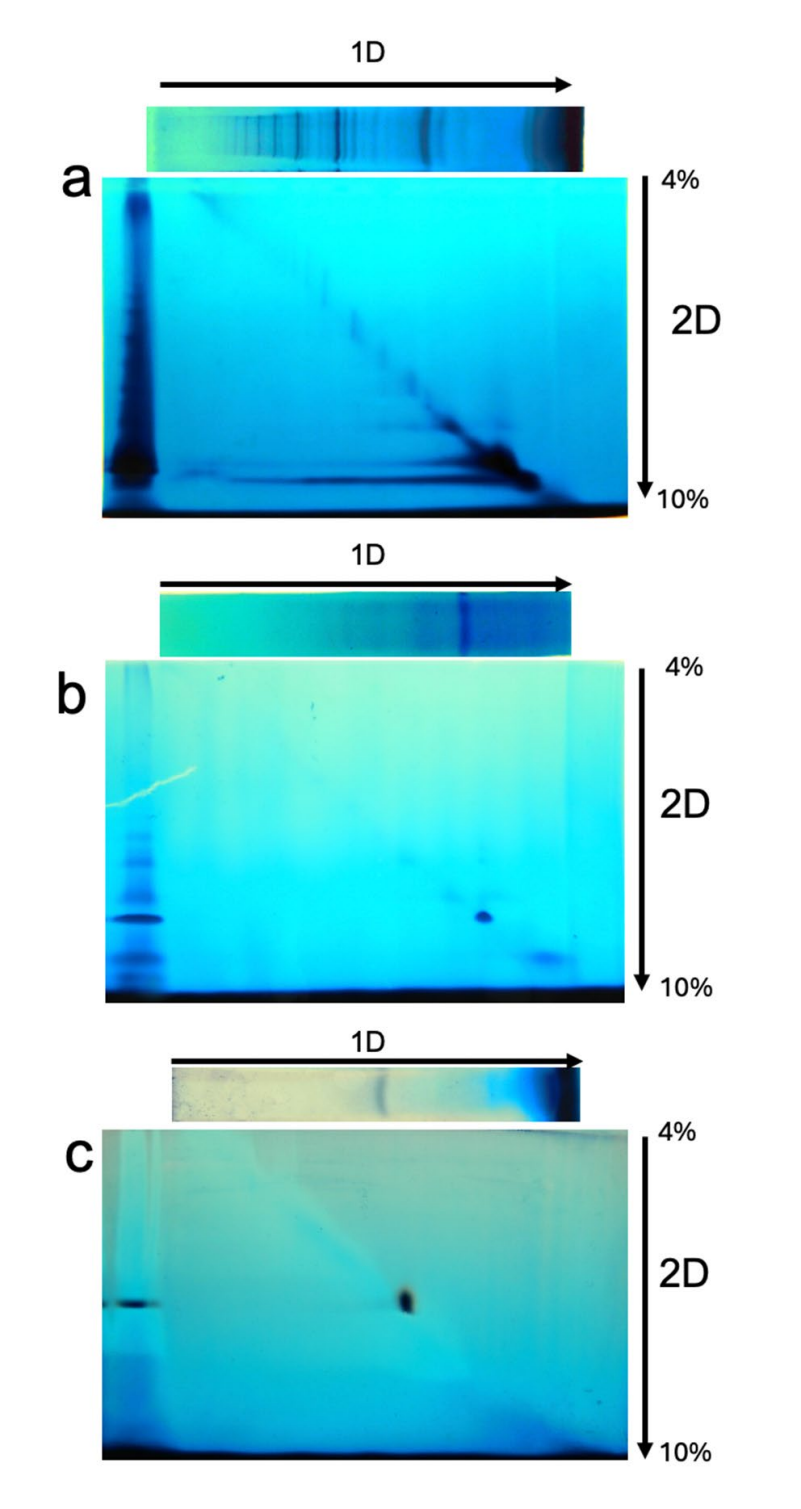
2D-BN-PAGE of digitonin-solubilized membranes from *B. licheniformis* grown in Nut medium previously resolved by 1D BN-PAGE. **a**) NADH dehydrogenase, **b**) NADPH dehydrogenase, and **c**) succinate dehydrogenase

Several bands of NADH dehydrogenase activity were detected in membranes from Nut- and MM-NH_4_-grown cells but not in the MM-CN-grown cells (Fig. 9), probably because of the low NADH-DH activity in cells grown in the presence of cyanide (Fig. 7). It seems that the activity in the gel appears when the specific activity of the NADH-DH is above certain limits (60–70 nmol min^− 1^ (mg protein)^−1^). Although we cannot discard the possibility that NADH-DH associates with other respiratory complexes or proteins, such as the *bc-caa*_*3*_ or the two quinol oxidases (*aa*_*3*_ and *bd*), the ladder banding pattern suggests the formation of homo-oligomers instead of the classic supercomplexes. Among the 5 NADH dehydrogenases found in the *B. licheniformis* genome and our proteomic study (Fig. 10; Table 2), only the enzyme with the accession number A0A1Y0YFU0 was associated with the high molecular weight bands containing NADH-DH activity (Bands 2 to 4 in Fig. 10; Table 2). The other NADH-DH were found near the bottom of the gel (Band 1 of Fig. 10; Table 2). Interestingly, the NADH-DH activity in *B. subtilis* appeared as a single band near the bottom of the gel, indicating that this enzyme could not associate with other proteins (García Montes De Oca et al. 2012; Picón Garrido et al. 2022) or form homo-oligomers. Gaining a better understanding of the NADH-DH aggregation mechanism in *B. licheniformis* will require the cloning, expressing, and purifying the enzyme.

The NADPH-DH activity in the gel was observed only in membranes obtained from Nut-grown cells (Fig. 9b). The protein’s molecular mass was around 173 kDa, suggesting a dimeric structure for the protein. In agreement with the competition plot (Fig. 8), NADH and NADPH dehydrogenase activities did not co-localize in the gel, further confirming the presence of two enzymes with different specificities for the coenzymes.

Membranes from Nut- and MM-NH_4_-grown cells, but not from MM-CN-grown cells, showed succinate dehydrogenase activity in the gel (Fig. 9c). The SDH migrated with a molecular mass of 375 kDa, much higher than the predicted from the genome (115 kDa). In agreement with the enzyme activity assays (Fig. 7), the in-gel activity for SDH was not detected in the MM-CN condition. It is worth mentioning that the bands containing the NADH, NADPH, and succinate dehydrogenase appear within 1 h incubation of the gel slices in the specific reaction mixtures. On the other hand, the cytochrome c oxidase band was observed only in the Nut condition after 48 h incubation of the gel slice with the reaction mixture, indicating a low activity of the *caa3* complex.

### 2D native electrophoresis

Digitonin is a mild detergent that extracts respiratory complexes and supercomplexes from the inner mitochondrial membrane while preserving the structural integrity of the supercomplexes (Cogliati et al. 2021). In contrast, the detergent DDM is a stronger agent that disassembles the supercomplexes’ components while maintaining the structure of the individual complexes (Lenaz et al. 2016).

Consequently, we solubilized the complexes using digitonin, performed a 1D BN-PAGE electrophoresis, incubated the gel slice with 0.02 or 0.1% DDM, and conducted a 2D BN-PAGE to separate the components of the respiratory complexes (Schagger et al. 1994). If the low molecular weight NADH dehydrogenase dissociates from the aggregates, its activity will appear near the bottom of the gel. Conversely, if the aggregates exhibit high stability, their migration will correspond to the molecular weight of the complex. If multiple stable complexes with NADH-DH activity exist, the activity will appear as a diagonal on the gel. Figure 10 shows that a significant portion of the NADH-DH activity dissociated from the high molecular weight complexes, likely corresponding to the dimeric form of the protein, and migrated to the bottom of the gel. Nonetheless, another fraction of the NADH-DH population remained as high molecular weight complexes and were distributed along the diagonal of the gel, suggesting strong interactions between the NADH-DH monomers.

**Fig. 11:**
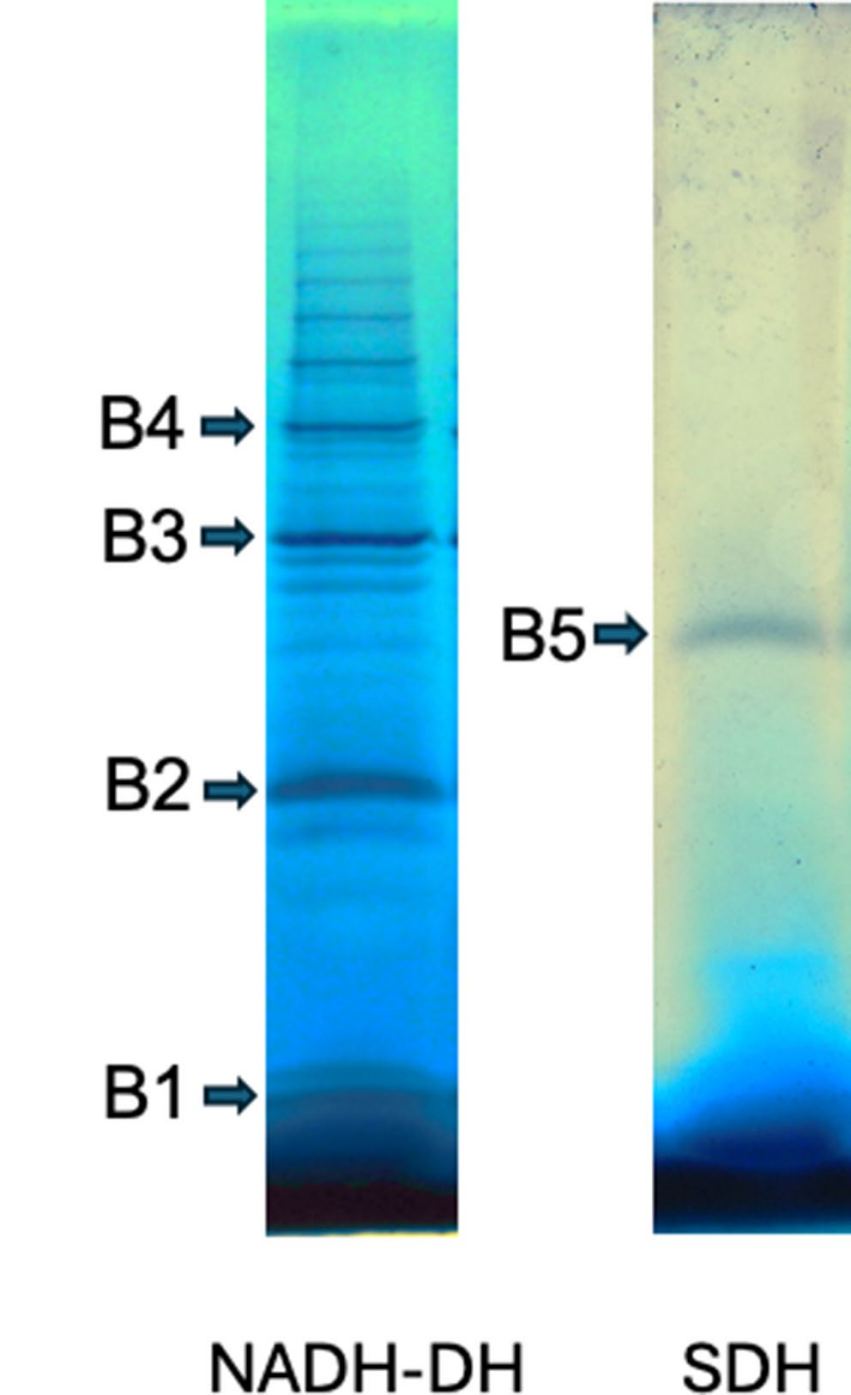
1D-BN-PAGE of *B. licheniformis* membrane proteins. Bands B1 to B5 were analyzed by LC/MS-MS

For NADPH dehydrogenase, its migration indicates the high stability of the putative dimer. Interestingly, a less intense spot closer to the end of the gel also shows NADPH dehydrogenase activity, implying the existence of at least two enzymes capable of using NADPH as a substrate. Previous studies (García Montes De Oca et al. 2012) and our proteomic investigation (Band 5 of Fig. 11; Table 2) showed that the putative complex comprising the succinate dehydrogenase and the nitrate reductase was robust enough to withstand the high concentration of DDM.

In agreement with the studies in *B. subtilis* (Winstedt and Von Wachenfeldt 2000), our proteomic study (Table 2) revealed the presence of three terminal oxidases in *B. licheniformis*: the bc-caa3 and the two quinol oxidases, bd and aa3. Also, we did not find evidence for the presence of the bb’ oxidase (Azarkina et al. 1999). The experimental results showed that in *B. licheniformis* grown in the presence of acetate, the predominant terminal oxidases were bd and aa3, while the activity of bc-caa3 was negligible. This was consistent with the oxygen consumption results, where the bc-caa3 inhibitor antimycin A had no effect. Furthermore, when cells were cultured in the Nut medium, cyanide substantially reduced respiratory activity (90%), inhibiting the aa3 oxidase. In contrast, cyanide had a limited effect on cells grown in a minimal medium with acetate, indicating that the bd oxidase was the predominant form under this condition.

**Table 2:** LC-MS/MS analysis of 1D BN-PAGE spots (1–5) identified in *B. Licheniformis* membranes from nut-grown cells

Spot	Protein	Respiratory chain complex	UniProt accession	kDa	Aa
B1	Cytochrome c551	Cytochrome c	Q65EC8	11	108
	NAD(P)/FAD oxidoreductase	NDH-2	A0A1Y0YFU0	42	392
	NAD(P)/FAD oxidoreductase	NDH-2	A0A1Y0YJN6	44	403
	NAD(P)/FAD oxidoreductase	NDH-2	A0A415ITK0	46	424
	NADPH dehydrogenase	NDH-2	A0A7Y6P2J4	40	356
	NAD(P)/FAD oxidoreductase	NDH-2	A0A415J766	39	355
B2	NAD(P)/FAD oxidoreductase	NDH-2	A0A1Y0YFU0	42	392
	Nitrate reductase (quinone)	NQR	A0A415JFE2	138	1228
	Cytochrome bd-I ubiquinol oxidase subunit 1	bd	A0A415J941	52	468
	Cytochrome bd-I ubiquinol oxidase subunit 2	bd	A0A415J974	37	338
	Cytochrome c551	Cytochrome c	Q65EC8	11	108
B3	Fumarate reductase flavoprotein subunit	SDH	A0A1Y0YIG7	65	587
	Fumarate reductase iron-sulfur subunit	SDH	A0A5Q3BS85	28	254
	Cytochrome c oxidase subunit 4B	caa3	A0A1Y0YHC3	12	109
	Cytochrome c oxidase subunit 2	caa3	A0A415J1P1	40	355
	Cytochrome c551	Cytochrome c	Q65EC8	11	108
	Quinol oxidase subunit 2	aa3	A0A1Y0Y193	36	322
	Cytochrome bd-I ubiquinol oxidase subunit 1	bd	A0A415J941	52	468
	Cytochrome bd-I ubiquinol oxidase subunit 2	bd	A0A415J974	37	338
	Menaquinol-cytochrome c reductase cytochrome b/c subunit	bc	A0A1Y0YTG6	28	255
	NAD(P)/FAD oxidoreductase	NDH-2	A0A1Y0YFU0	42	392
B4	Fumarate reductase flavoprotein subunit	SDH	A0A1Y0YIG7	65	587
	Fumarate reductase iron-sulfur ubunit	SDH	A0A5Q3BS85	28	254
	Cytochrome c oxidase subunit 2	caa3	A0A415J1P1	40	355
	Quinol oxidase subunit 2	aa3	A0A1Y0Y193	36	322
	Quinol oxidase subunit 3	aa3	A0A1Y0YLA8	22	204
	Cytochrome b6-f subunit of the iron-sulfur complex	bc	A0A1J6GLT1	19	168
	Cytochrome C	Cytochrome c	A0A1Y0YRB6	12	119
	Cytochrome c551	Cytochrome c	Q65EC8	11	108
	Cytochrome bd-I ubiquinol oxidase subunit 1	bd	A0A415J941	52	468
	Cytochrome bd-I ubiquinol oxidase subunit 2	bd	A0A415J974	37	338
	NAD(P)/FAD oxidoreductase	NDH-2	A0A1Y0YFU0	42	392
	NAD(P)/FAD oxidoreductase	NDH-2	A0A1Y0YJN6	44	403
B5	Fumarate reductase flavoprotein subunit	SDH	A0A1Y0YIG7	65	587
	Fumarate reductase iron-sulfur ubunit	SDH	A0A5Q3BS85	28	254
	Quinol oxidase subunit 1	aa3	A0A415J8U4	73	649
	Quinol oxidase subunit 2	aa3	A0A1Y0Y193	36	322
	Quinol oxidase subunit 3	aa3	A0A1Y0YLA8	22	204
	Nitrate reductase (quinone)	NQR	A0A415JFE2	138	1228
	Nitrate reductase beta subunit	NQR	A0A415JFG8	55	489
	Menaquinol-cytochrome c reductase cytochrome b/c subunit	bc	A0A1Y0YTG6	28	255
	Cytochrome c oxidase subunit 2	caa3	A0A415J1P1	40	355
	Cytochrome b6-f subunit of the iron-sulfur complex	bc	A0A1J6GLT1	19	168
	Cytochrome c551	Cytochrome c	Q65EC8	11	108
	NAD(P)/FAD oxidoreductase	NDH-2	A0A1Y0YFU0	42	392

## Conclusion

*B. licheniformis* can carry out the assimilation of cyanide as a nitrogen source and perform aerobic respiration in the presence of this toxic compound. *B. licheniformis* changes its respiratory activity depending on the culture medium. When cells were grown in the presence of cyanide, this compound increased oxygen consumption in cells but not in membranes, indicating the presence of cytosolic enzymes involved in the cyanide metabolism. *B. licheniformis* membranes contain NADH and NADPH dehydrogenases, and one NADH dehydrogenase (A0A1Y0YFU0) can form various aggregates, probably homo-oligomers. SDH was not detected in cells growing in MM-CN, either by in-gel activity or measurements of the specific activity in membranes. Oxygen consumption in the presence of inhibitors indicates that the quinol oxidase aa3 is the main oxidase in *B. licheniformis* cultured in acetate as the carbon source. In this work, we showed that the terminal oxidase bd confers resistance to this bacterium for cyanide, an important mechanism for its use in biotechnological bioremediation processes. In addition, we demonstrated that oxygen is a crucial factor to consider when carrying out projects to treat cyanide-contaminated wastewater since the oxidative metabolism of cyanide demands large amounts of oxygen. For bioremediation applications, the genes responsible for the transport and assimilation of cyanide in *Bacillus licheniformis* could be identified. Subsequently, this organism could be genetically modified to overexpress proteins associated with cyanide metabolism, enhancing its capacity to degrade this compound. Additionally, overexpressing proteins involved in ATP synthesis, such as the ATP synthase and the bd terminal oxidase, could further improve the organism’s ability to address environmental cyanide pollution. This approach could constitute an effective and sustainable strategy to mitigate cyanide contamination, contributing to ecological health and environmental security.

## Data Availability

No datasets were generated or analysed during the current study.
